# Early administration of fibrinogen concentrate in patients with polytrauma with thromboelastometry suggestive of hypofibrinogenemia: A randomized feasibility trial

**DOI:** 10.6061/clinics/2021/e3168

**Published:** 2021-10-28

**Authors:** Lucas Siqueira de Lucena, Roseny dos Reis Rodrigues, Maria José Carvalho Carmona, Francisco José Diniz Noronha, Helenode Paiva Oliveira, Natalia Martins Lima, Rodrigo Brandão Pinheiro, Wallace Andrino da Silva, Alexandre Biasi Cavalcanti

**Affiliations:** I Programa de Pos-graduacao em Anestesiologia, Ciencias Cirurgicas e Medicina Perioperatoria, Faculdade de Medicina FMUSP, Universidade de Sao Paulo, Sao Paulo, SP, BR.; II Departamento de Anestesiologia, Hospital Universitario Walter Cantidio, Fortaleza, CE, BR.; III Departamento de Anestesiologia, Hospital Universitario Onofre Lopes, Natal, RN, BR.; IV Instituto de Pesquisa Hcor, Sao Paulo, SP, BR.

**Keywords:** Multiple Trauma, Coagulopathy, Fibrinogen, Thromboelastometry

## Abstract

**OBJECTIVE:**

To evaluate the clinical effects of early administration of fibrinogen concentrate in patients with severe trauma and hypofibrinogenemia.

**METHODS:**

We conducted an open randomized feasibility trial between December 2015 and January 2017 in patients with severe trauma admitted to the emergency department of a large trauma center. Patients presented with hypotension, tachycardia, and FIBTEM findings suggestive of hypofibrinogenemia. The intervention group received fibrinogen concentrate (50 mg/kg), and the control group did not receive early fibrinogen replacement. The primary outcome was feasibility assessed as the proportion of patients receiving the allocated treatment within 60 min after randomization. The secondary outcomes were transfusion requirements and other exploratory outcomes. Randomization was performed using sequentially numbered and sealed opaque envelopes. ClinicalTrials.gov: NCT02864875.

**RESULTS:**

Thirty-two patients were randomized (16 in each group). All patients received the allocated treatment within 60 min after randomization (100%, 95% confidence interval, 86.7%-100%). The median length of intensive care unit stay was shorter in the intervention group (8 days, interquartile range [IQR] 5.75-10.0 *vs*. 11 days, IQR 8.5-16.0; *p*=0.02). There was no difference between the groups in other clinical outcomes. No adverse effects related to treatment were recorded in either group.

**CONCLUSION:**

Early fibrinogen replacement with fibrinogen concentrate was feasible. Larger trials are required to properly evaluate clinical outcomes.

## INTRODUCTION

Uncontrolled hemorrhage and its repercussions are the leading preventable causes of death in emergency scenarios (1). Several independent studies have shown that 25% of patients with severe trauma arrive at emergency departments with hemodynamic deficits and acute traumatic coagulopathy (ATC) (2). Despite advances, the role of fibrinogen in this coagulopathy and the potential benefits of its early replacement have not been fully evaluated.

Acquired fibrinogen deficiency occurs during trauma (3). Fibrinogen deficiency may occur in the early stages of fluid and blood product replacement (4) and may be aggravated by hemodilution, factor consumption, acidosis, and hypothermia (5). Fibrinogen is the most vulnerable coagulation factor and is the first to reach very low levels after trauma (4). A low fibrinogen concentration is common among patients with severe trauma and is associated with increased blood loss and/or transfusion (6). A low fibrinogen level is an independent risk factor for death in patients requiring massive transfusion (3).

A recent study suggests that viscoelastic hemostatic assays detect acute coagulopathy more accurately and substantially faster than conventional laboratory examinations (7). The result of FIBTEM-ROTEM^®^ correlates well with conventional laboratory measurements of serum fibrinogen concentration, can be used as a marker of ATC, and can predict the need for massive transfusion (8,9). Studies have suggested using FIBTEM as a tool to reduce blood transfusions in several clinical situations (10,11), including polytrauma with severe bleeding (12). To quickly assess ATC, thromboelastometry parameters at the fifth minute (A5) can be used, as they have good correlation with the maximum clot firmness (MCF) (8).

Fibrinogen concentrate (FC) is a blood product derived from a pool of human plasma after pasteurization, nanofiltration, and viral inactivation. Possible benefits including immunomodulation, reduction of infectious risks, and organic dysfunctions have been suggested, but also questioned (13-15). Significant gains have been achieved in restoring serum fibrinogen levels and reestablishing hemostasis in patients with congenital (16) and acquired hypofibrinogenemia (17,18), especially patients with obstetric hemorrhage (19), bleeding associated with ATC (8,20), and cardiovascular surgeries (21). Thromboembolic complications are among the main concerns associated with the administration of FC. However, several studies (22-26) suggest that there is no increase in thromboembolic events in patients treated with FC.

Thus, administration of FC to patients with severe trauma is promising, but its clinical effects remain to be evaluated in randomized clinical trials (RCTs). We intend to conduct a large RCT to evaluate the clinical effects of early fibrinogen replacement using FC in patients with severe trauma and signs suggestive of hypofibrinogenemia (FIBTEM A5 ≤9 mm). However, it is necessary to evaluate the feasibility of conducting a large RCT in this scenario. This feasibility study is presented in this paper.

## MATERIAL AND METHODS

### Design and ethical aspects

This randomized, non-blinded study was designed to evaluate the feasibility of early administration of FC in patients with severe trauma and evidence of fibrinogen deficiency on thromboelastometry (FIBTEM-ROTEM^®^). Early intervention was defined as intervention performed up to 60 min after fulfilling the inclusion criteria, as described in the literature (27). The study was conducted between December 2015 and January 2017 at the Emergency Surgery Service of the Clinical Surgical Division of the Hospital das Clínicas da Faculdade de São Paulo (HC-FMUSP), in the emergency, surgery, and intensive care units (ICU). This study was approved by the Ethics Committee for Analysis of Research Projects of HC-FMUSP and was registered at ClinicalTrials.gov (NCT02864875).

Written informed consent was obtained from the patients or their representatives. In cases in which this was not possible, an independent physician not involved in this research was responsible for signing the free informed consent terms, allowing the patient to be included in the study. As soon as possible, a new informed consent was obtained from the patient’s appropriate representative by the investigator in charge.

Data were collected in the emergency department, operating room (OR), ICU, and wards. Patients were followed up until discharge or death. This manuscript was written according to the guidelines of the Consolidated Standards of Reporting Trials (CONSORT) adapted for pilot and feasibility studies (28).

### Patients

The inclusion criteria of the study were as follows: patients aged 18-80 years admitted to the emergency department with severe trauma (index of shock severity [ISS] ≥15), hypotension (systolic blood pressure <90 mmHg), tachycardia (heart rate >100 bpm), and no indication for inclusion in the institutional massive transfusion protocol (MTP). Patients eligible for MTP were excluded because early replacement using only fibrinogen would not be feasible. Patients with all the above inclusion criteria were subjected to thromboelastometry and were included if qualitative hypofibrinogenemia was diagnosed (FIBTEM A5 ≤9 mm). The cutoff of FIBTEM A5 ≤9 mm was chosen based on previous studies demonstrating good association with the presence of ATC and the necessity for MTP (29).

Blood samples for thromboelastometry were collected by a research nursing technician in the emergency room. Samples were immediately subjected to thromboelastometry in the OR where the device was located. The examination was performed by a trained technician. The results were added to the research data and communicated verbally to the assistant physician. FC was obtained from the OR pharmacy.

The exclusion criteria were as follows: pregnant patients according to admission data, patients with previously known coagulation disorders, use of anticoagulant drugs and/or previous antiplatelet drugs (with the exception of acetylsalicylic acid), patients with a previous history of thromboembolic events, history of cardiorespiratory arrest at trauma scene, patients transferred from another service, time between trauma and admission >6 h, and patients with exclusively cranioencephalic trauma.

### Randomization

A randomized allocation list for the experimental and control groups (1:1 allocation rate) was generated by a statistician using appropriate software (www.randomization.com). Blocks of size 16 were used in this study. Treatment allocation was performed using sequentially numbered sealed opaque envelopes immediately after the FIBTEM A5 results. The envelopes were prepared by a researcher who did not participate in patient inclusion.

### Interventions

The experimental group received a single dose of 50 mg/kg of body weight of FC immediately after allocation. This was the mean replacement dose in previous trials (30). The presentation of FC was one gram per vial (Haemocomplettan^®^ P, CSL Behring, Marburg-Germany). It was administered by a research physician within a period of <5 min, as described in other clinical trials (31,32). No patient in the control group received fibrinogen within 60 min after fulfilling clinical inclusion criteria. After the initial intervention, decisions regarding the transfusion of blood products were left to the attending physician. Blood transfusions followed an institutional protocol (33).

Both study groups received a 1g loading dose of tranexamic acid, followed by continuous infusion of 1g over 8h (if they were within a 3h period from the trauma), and both groups were optimized for acidosis, hypocalcemia, and hypothermia management.

### Outcomes

The primary outcome was feasibility, defined as the proportion of patients treated according to the allocation. The intervention group should receive FC at a dose of 50 mg/kg body weight within 60 min after randomization. The control group should not receive fibrinogen replacement within 60 min after randomization.

Exploratory secondary outcomes were: blood loss through drains in the first 48h after hospital admission and during hospitalization; amount of packed red blood cells transfused in the first 48h after hospital admission, in the OR, in the ICU, and during hospitalization; amount of plasma transfused in the first 48h after hospital admission, in the OR, in the ICU, and during hospitalization; amount of platelets (apheresis) transfused in the first 48h after hospital admission, in the OR, in the ICU, and during hospitalization; amount of cryoprecipitate transfused in the first 48h after hospital admission, in the OR, in the ICU and during hospitalization; rate of thromboembolic events with clinical manifestation in the first two weeks of hospitalization; recurrent bleeding requiring surgery during hospitalization; number of ventilator-free days during hospitalization; number of vasopressor-free days during hospitalization; length of ICU and hospital stay; sequential organ failure assessment (SOFA) score on the first, fifth and seventh day after admission to the ICU; and number of in-hospital deaths.

Data from all outcome variables were obtained from the patients’ physical and/or electronic records.

### Statistical analysis

In this pilot study, a sample size of 32 patients was defined by convenience. With this sample size, assuming that 95% of the cases would adhere to the allocated treatment, we would have a 95% confidence interval (CI) of amplitude near 15%.

For continuous outcomes with normal distribution, we presented the mean difference, 95% CI, and *p*-value calculated using the t-test. For continuous outcomes with asymmetric distribution, we performed the Mann-Whitney test. We evaluated whether the continuous data were normally distributed using the Shapiro-Wilk test and visual histogram analysis.

Categorical variables are presented as absolute (n) and relative (%) frequencies. We estimated the effect of intervention on categorical secondary outcomes using the risk ratio, 95% CI, and chi-square test. Statistical significance was set at *p*<0.05. No adjustments were made to the *p*-values (or levels of significance) for the multiple hypothesis tests. Therefore, the interpretation of the effects on multiple secondary outcomes was exploratory. The data were analyzed using the program R.

## RESULTS

### Patient characteristics and adherence to treatment

**Figure 1:**
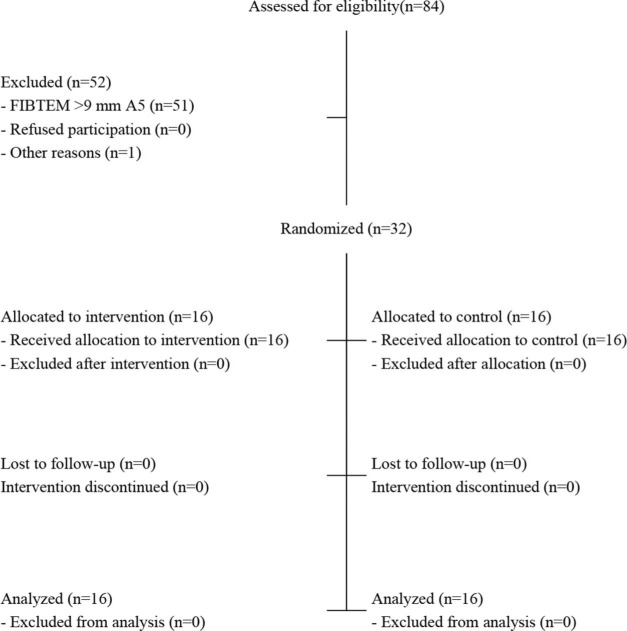
Patient flow in the study. Source: CONSORT, 2010.

A total of 84 patients were assessed for eligibility, and 52 were excluded (Figure 1). Among these patients, 51 were excluded because they did not meet the eligibility criteria, and one had sudden worsening and died before randomization. Finally, 32 patients were randomized — 16 in the control group and 16 in the experimental group. None of the patients refused to participate after randomization. All randomized patients were analyzed according to their allocated groups.

Patient recruitment was initiated in December 2015 and was completed in January 2017. Patients were followed up throughout hospitalization with directed data collection during the first 15 days of their hospital stay and were followed up until discharge or death. The study was finalized after the planned number of patients was reached.

Table 1 shows the characteristics of the patients on admission to the emergency department. Both groups presented demographic similarities, with an average age in the fifth decade of life. There was a greater number of patients with traumatic brain injury (7 of 16 and 1 of 16 patients, respectively; *p*=0.01) in the intervention group. The mean serum fibrinogen dosage (mg/dL) was lower in the control group than that in the intervention group (107.5±61.6 and 143.5±53.5, respectively), but the difference was not statistically significant (p=0.09) and the qualitative fibrinogen evaluations performed through FIBTEM MCF were similar (7.2±2.4 and 6.9±3.3 mm, respectively). Other clinical and laboratory characteristics were similar in both groups.

### Primary outcome

All patients in the intervention group received FC at 50 mg/kg of body weight within 1h after randomization. None of the patients in the control group received fibrinogen within the first hour after randomization. Therefore, 100% of the patients were administered the allocated treatment (95% CI, 86.7% to 100%). The mean time from inclusion based on clinical criteria until FIBTEM execution was 31.2±8.3 min and 31.8±10.7 for the control and intervention groups, respectively (with minimum and maximum times of 10 min and 50 min, respectively, in both groups). The time from the FIBTEM A5 test result and randomization was ≤5 min.

### Exploratory secondary outcomes

In the intervention and control groups, 14/16 (87.5%) and 15/16 (93.8%; *p*=1.00) patients underwent surgery, respectively. In the OR, the mean serum fibrinogen level was higher in the intervention group than that in the control group (190.4±85.5 *vs.* 130.2±51.1; *p*=0.04). There were no statistically significant differences in any of the other OR variables.

Regarding clinical and laboratory variables at ICU admission, the serum pH of the control group was lower than that of the intervention group (7.2±0.1 *vs.* 7.3±0.1; *p*=0.009) and the heart rate was lower in the intervention group than that in the control group (94±12 *vs.* 110±19; *p*=0.01). There were no statistically significant differences in any of the other variables. All patients were admitted to the ICU.

There was a statistically significant difference in the secondary exploratory outcome length of ICU stay between the intervention group (median 8, interquartile range [IQR] 5.75-10.0) and the control group (median 11, IQR 8.5-16.0; *p*=0.02). There were no statistically significant differences in any other secondary exploratory outcomes.

For the assessment of variables involving blood loss through drains, only patients who had drains after the surgical procedure were included (10 patients in the control group and 12 patients in the intervention group). Median blood loss (in mL) through drains during the first 48h after hospital admission (*p*=0.41) and during hospital stay (*p*=0.84) are shown in Table 2. There were no statistically significant differences between the intervention and control groups.

The transfused blood components (units) are listed in Table 2. There were no statistically significant differences between the intervention and control groups in packed red blood cells (*p*=0.548), fresh plasma (*p*=0.437), platelets (*p*=0.495), and cryoprecipitate (*p*=0.284).

Other exploratory clinical outcomes are shown in Table 3. No subgroup analyses or adjusted analyses were performed. No damage or undesirable effects were observed.

## DISCUSSION

This trial demonstrated that it is feasible to carry out a larger randomized trial to compare early replacement of fibrinogen at 50 mg/kg body weight with no early replacement of fibrinogen (within 60 min) in patients with severe trauma (ISS >15), hypotension (systolic blood pressure <90 mmHg), tachycardia (heart rate >100 bpm), and signs of qualitative hypofibrinogenemia (FIBTEM A5 ≤9).

The mean time from inclusion based on clinical criteria until FIBTEM execution was approximately 30 min in both groups. Immediately after allocation, an investigator performed the treatment in the intervention group. Our results are similar to those of a recent study, which showed that it was possible to administer FC within 50 min after arrival to the emergency department in 95% of patients allocated (34).

Most previous studies evaluated the time to start fibrinogen replacement from hospital admission and exclusively used clinical parameters as inclusion criteria (27,34,35). In this study, we measured the time between meeting clinical inclusion criteria and administration of FC because some patients do not meet the eligibility criteria upon arrival at the emergency department, but do so later. A qualitative hypofibrinogenemia parameter was added to the clinical criteria to select patients with true fibrinogen deficit who would have a greater theoretical benefit from the proposed intervention, as observed in a recent study (36).

The use of viscoelastic assays for rapid and reliable detection of patients with ATC has been reported in the literature (37). FIBTEM can be used independently of other thromboelastometric curves as a marker of ATC, a predictor of massive transfusion requirement that correlates well with the conventional measurement of serum fibrinogen concentration (38,39). We use only one thromboelastometric curve due to the limited resources.

Fibrinogen is often administered in the later stages of transfusion therapy (27). It is replaced using cryoprecipitate and FC in most major trauma centers, and according to a recent review, one should not be preferred over the other (40). Agility in treating hypofibrinogenemia in the context of this study may contribute to bleeding control, coagulopathy resolution, and reduction in transfusion requirement (8,41,42). Recent evidence has shown that it is possible to perform early replacement with cryoprecipitate (27), as has already been demonstrated with FC (34). FC is a blood product derived from a pool of human plasma after pasteurization, nanofiltration, and viral inactivation, and several studies have demonstrated its safety and efficacy (25). In this study, no adverse reactions related to administration of FC were observed. No clinical evidence of thrombotic complications was found in either group. However, this variable was difficult to evaluate because of the small number of patients involved.

As recently demonstrated, fibrinogen levels are elevated by administration of cryoprecipitate or FC even in patients with severe bleeding (27,34,35). Over time, these levels tend to equilibrate, regardless of the initial replacement, suggesting the existence of an important fibrinogen concentration regulating system that may also protect against late thromboembolic complications (34).

There were no statistically significant differences in the need for transfusion of blood components or blood loss among the groups. There was a statistically significant difference in the secondary exploratory outcome length of ICU stay between the intervention and control groups. However, this might well have been a chance finding due to the multiplicity of hypothesis testing.

The present study suggests a different and feasible approach to the inclusion criteria (using a thromboelastometric parameter, FIBTEM A5) with possible selection benefits. There is an ongoing study using a similar approach (36). Although we used an intervention with a fixed dose (50 mg/kg), FIBTEM A5 can be used to guide fibrinogen replacement doses, as suggested in another clinical study (27).

This study had several limitations. Exploratory secondary outcomes should be considered with caution because the study had low power to assess clinical outcomes, and there was an increased probability of spurious associations due to the multiplicity of hypothesis tests. It was not possible to blind the intervention and create a placebo package due to staff shortage in the pharmacy department, and it was also not possible to hire additional staff because of limited study funding resources. Nevertheless, it was observed that nonblinding simplified the intervention process and shortened the treatment administration time. Although there is an institutional transfusion guideline emphasizing the restrictive and rational use of blood components, knowledge of the assigned prescribing physicians could have created a bias.

Some clinical trials that evaluated early replacement of fibrinogen excluded patients with severe head trauma classified as unsalvageable (35) or catastrophic (34). Similarly, others excluded patients who were assessed as having injuries incompatible with life (27,36). The absence of an exclusion criterion that removes patients with injuries associated with very high mortality may have interfered with the exploratory clinical outcomes and should therefore be added to the design of future RCTs.

FC can be easily stored, reconstituted, and administered. Brazil is a country with continental dimensions, where logistics for training of personnel and renewing blood banks are extremely difficult. The possibility of preparing ahead of time to treat a coagulation disorder that can strongly impact patient outcomes is of paramount importance and should be studied with great care. At present, there are no high-quality studies that support the application of FC.

## CONCLUSION

The results of this feasibility study in conjunction with other ongoing and concluded clinical trials will facilitate the design of large RCTs with sufficient statistical power to assess important clinical outcomes in the transfusional management of severe trauma and major bleeding.

## AUTHOR CONTRIBUTIONS

Lucena LS was responsible for the literature search, study design, data collection and interpretation, manuscript writing and critical revision. Rodrigues RR, Noronha FJD and Oliveira HP were responsible for the literature search, study design, and data collection. Carmona MJC was responsible for the study design, and critical revision. Lima NM, Pinheiro RB and Silva WA were responsible for data interpretation, manuscript writing and critical revision. Cavalcanti AB was responsible for the literature search, study design, data analysis and interpretation, manuscript writing and critical revision.

## Figures and Tables

**Table 1:** Characteristics of patients on admission to the emergency room.

Characteristics	Control (n=16)	Intervention (n=16)
Age - years, average±SD	40.2±15.7	44±16.3
Female sex, No. (%)	3 (18.8)	1 (6.2)
Weight - kg	75.2±14.5	77.9±9.6
Glasgow coma scale	13.6±2.5	11±4.6
Presence of TBI	1 (6.2)	7 (43.8)
Index of shock severity	29.8±7.2	34.3±7.4
ABC score (inclusion)	1.8±0.4	1.8±0.5
Shock index (inclusion)	1.6±0.4	1.6±0.3
FAST positive	5 (31.2)	4 (26.7)
Prehospital crystalloids - mL	1134.6±711.6	1250±755.9
FIBTEM A5*	6.06±1.8	5.43±2
FIBTEM MCF	7.2±2.4	6.9±3.3
FIBTEM ML	10.6±14.2	9.3±8
Serum fibrinogen - mg/dL†	107.5±61.6	143.5±53.5
Hemoglobin - g/dL	11.4±2.1	11.8±2.8
APT (INR)	1.7±1.3	1.3±0.2
aPTT (R)	14.5±15.3	14.5±15.3
Platelets - x1000/mm^3^	182.9±61.1	189.3±57.5
pH	7.3±0.1	7.3±0.1
Base excess - mmol/L	-8.3±4.3	-7.1±6.2
Lactate - mg/dL	43.9±27.6	33.3±34.6
Ionized calcium - mg/dL	4.3±0.3	4.4±0.7
Esophageal temperature - °C	35.7±0.4	35.7±0.5
Transportation time to hospital - min	42.5±51.1	39.1±15.8
Tranexamic acid within delta T- No. (%) ‡	13 (81.2)	12 (75)

TBI, traumatic brain injury; FAST, focused abdominal sonogram for trauma; MCF, maximum clot firmness; ML, maximum lysis; APT, activated prothrombin time; INR, international normalized ratio; aPTT, activated partial thromboplastin time; R, relative. pH: Hydrogen potential.

* A5 refers to the fifth minute from the beginning of the exam.

† Fibrinogen measured using the Clauss method.

‡ Delta T indicates the first three hours after trauma.

**Table 2:** Exploratory secondary outcomes reflecting bleeding and replacement of blood components.

Outcome	Control	Intervention	*p* value
Blood loss through drains in first 48h - mL, median (IQR)	560.0 (362.5-925.0)	287.5 (125.0-700.0)	0.41
No. of PC units transfused in first 48h - Median (IQR)	4.5 (2.0-8.0)	4.0 (3.25-5.25)	0.63
Fresh plasma units transfused in first 48h - Median No. (IQR)	1.5 (0.0-4.5)	0.0 (0.0-5.0)	0.41
Platelets (apheresis) units transfused in first 48h - Median No. (IQR)	0.0 (0.0-1.0)	0.0 (0.0-0.25)	0.61
Cryoprecipitate units transfused in first 48h - No. median (IQR)	0.0 (0.0-5.75)	0.0 (0.0-1.0)	0.28
Blood loss through drains - mL, median (IQR)	2415.0 (2285.0-3700.0)	1465.0 (547.5-3173.75)	0.85
No. of PC units transfused - Median (IQR)	6.5 (4.75-8.0)	6.5 (3.5-9.0)	0.55
No. of Fresh plasma units transfused - Median (IQR)	1.5 (0.0-4.25)	0.0 (0.0-5.0)	0.44
No. of Platelet units (apheresis) transfused - Median (IQR)	0.5 (0.0-1.0)	0.0 (0.0-0.25)	0.50
No. of Cryoprecipitate units transfused - Median (IQR)	0.0 (0.0-5.75)	0.0 (0.0-1.0)	0.28
No. of PC units transfused in the OR - Median (IQR)	2.0 (0.0-4.0)	2.0 (0.0-2.5)	0.51
No. of Fresh plasma units transfused in the OR - Median (IQR)	0.5 (0.0-4.0)	0.0 (0.0-2.0)	0.36
No. of Platelets (apheresis) units transfused in the OR - Median (IQR)	0.0 (0.0-1.0)	0.0 (0.0-0.0)	0.15
No. of Cryoprecipitate units transfused in the OR - Median (IQR)	0.0 (0.0-2.2)	0.0 (0.0-2.0)	0.58
No. of PC units transfused in the ICU - Median (IQR)	2.0 (0.7-3.0)	2.0 (0.75-3.25)	0.46
No. of Fresh plasma units transfused in the ICU - Median (IQR)	0.0 (0.0-0.0)	0.0 (0.0-0.0)	0.52
No. of Platelet (apheresis) units transfused in the ICU - Median (IQR)	0.0 (0.0-0.0)	0.0 (0.0-0.0)	0.66
No. of Cryoprecipitate units transfused in the ICU - Median (IQR)	0.0 (0.0-0.0)	0.0 (0.0-0.0)	0.33

PC: packed red blood cells; OR: OR; ICU: intensive care unit; IQR, interquartile range.

**Table 3:** Other exploratory clinical outcomes.

Outcome	Control	Intervention	Estimated Effect	*p* value
Thrombosis - No. of events / total number (%) (none)	16 (100)	16 (100)	-	-
Recurrent bleeding requiring surgery - No. of events / total number (%)	4 (25)	4 (25)	1 (0.36 - 2.03)	1.00
No. of Vasopressor-free days in the first 15 days, Median (IQR)	11.0 (7.7 - 13.2)	12.0 (3.75 - 12.25)	-	0.79
No. of Ventilator-free days in the first 15 days, Median (IQR)	12.0 (9.7 - 13.0)	10.0 (0.0 - 12.0)	-	0.22
Length of ICU stay - days, Median (IQR)	11.0 (8.5 - 16.0)	8.0 (5.75 - 1.0)	-	0.02
Length of hospital stay - days, Median (IQR)	18.5 (17.0- 21.0)	12.0 (10.0 - 22.0)	-	0.26
Intrahospital deaths - No. of events / total number (%)	3 (18.8)	5 (31.2)	0.69 (0.19 - 1.54)	0.46
SOFA score on the first day after admission to ICU, Median (IQR)	11.0 (9.0 - 12.0)	10.0 (7.25 - 11.75)	-	0.44
SOFA score on the fifth day after admission to ICU, Median (IQR)	7.0 (5.0 - 9.0)	7.0 (6.25 - 7.75)	-	0.95
SOFA score on the seventh day after admission to ICU, Median (IQR)	8.0 (7.5 - 8.5)	6.5 (3.25 - 9.75)	-	0.86

ICU - intensive care unit; IQR, interquartile range; SOFA - sequential organ failure assessment.
